# Targeting tumor microenvironment with antibody-guided IL-2 pro-cytokine promotes and rejuvenates dysfunctional CD8^+^ T cells

**DOI:** 10.1038/s41392-023-01463-y

**Published:** 2023-07-12

**Authors:** Xue Wang, Longchao Liu, Tao Yue, Zhichen Sun, Joonbeom Bae, Kuo-Fu Tseng, Anli Zhang, Jian Qiao, Yang-Xin Fu

**Affiliations:** 1 Department of Pathology, University of Texas Southwestern Medical Center, Dallas, TX 75235 USA; 2 CAS Key Laboratory of Pathogenic Microbiology and Immunology, Institute of Microbiology, Chinese Academy of Sciences, Beijing, 100101 China; 3 Aetio Biotherapy, Dallas, TX 75247 USA; 4 Department of Pharmacology, Harold C. Simmons Comprehensive Cancer Center, University of Texas Southwestern Medical Center, Dallas, TX 75390 USA; 5 Lyda Hill Department of Bioinformatics, University of Texas Southwestern Medical Center, Dallas, TX USA; 6 Department of Basic Medical Sciences, School of Medicine, Tsinghua University, Beijing, 100084 China

**Keywords:** Tumour immunology, Cancer

**Dear Editor**,

CLDN18.2 (CLDN), a member of tight junction protein family, is strictly limited to express on differentiated epithelial cells of the gastric mucosa and abnormal overexpression has been found in many cancers, especially in digestive system malignancies.^1^ Those features make CLDN a potential therapeutic target. However, monoclonal antibody targeting CLDN induce limited antitumor immune responses in clinical trials and fusion of strong immunomodulators might be needed to enhance its efficacy. High dose IL-2 activates tumor infiltrating lymphocytes (TILs), but the severe toxicity and poor tumor targeting limits its use.^2^

We first discovered that the abundance of CD8^+^ T cells and expression level of IL-2 in the tumor microenvironment (TME) were associated with better survival in several human cancers (Supplementary Fig. 1a, b), indicating that endogenous IL-2 might contribute to the infiltration and antitumor effect of CD8^+^ T cells. Indeed, when IL-2 signaling was blocked by anti-IL2Rβ (Supplementary Fig. 2a), the total number of T cells, the absolute number and percentage of CD8^+^ T cells were dramatically decreased (Supplementary, Fig. 2b–d). Meanwhile, the tumor grew much faster in treated-group (Supplementary Fig. 2e), suggesting sufficient IL-2 signaling is important for TILs-mediated antitumor immune response. We proposed that targeting tumor-activated IL-2 by anti-CLDN antibody could lead to more effective TILs with reduced toxicity.

We evaluated the characteristic of CLDN-ProIL2 in vitro and found that CLDN-ProIL2 can specifically bind to MC38-CLDN tumor cells with similar affinity to CLDN-Fc (Supplementary Fig. 3a, b). The binding affinity of Pro-IL2 was effectively blocked to a similar level as hIgG (Supplementary Fig. 3c). Meanwhile, IL-2 activity of CLDN-ProIL2 can be restored after MMPs digestion (Supplementary Fig. 3d).

To explore whether CLDN targeting is necessary for Pro-IL2 in vivo to act efficiently, we compared the antitumor efficacy of different constructs. Pro-IL2 has no anti-tumor activity, whereas CLDN-ProIL2 and CLDN-IL2 exhibited a superior antitumor efficacy in MC38-CLDN tumor model (Fig. 1a). However, CLDN-IL2 also induced a more severe systemic toxicity with dramatic body weight loss (Supplementary Fig. 4a), poor survival (Supplementary Fig. 4b) and a higher concentration of cytokines in the serum (Fig. 1b). Additionally, the percentage of NK cells and CD8^+^ T cells in the peripheral blood were dramatically increased (Supplementary Fig. 4c, d). In contrast, mice were tolerant to CLDN-ProIL2 with better survival. Moreover, CLDN-IL2 also trigger high level of alanine aminotransferase (ALT) and aspartate aminotransferase (AST) (Supplementary Fig. 4e, f). These results strongly suggest the toxicity induced by IL-2 was well-sealed by CLDN-ProIL2 design.

**Fig. 1:**
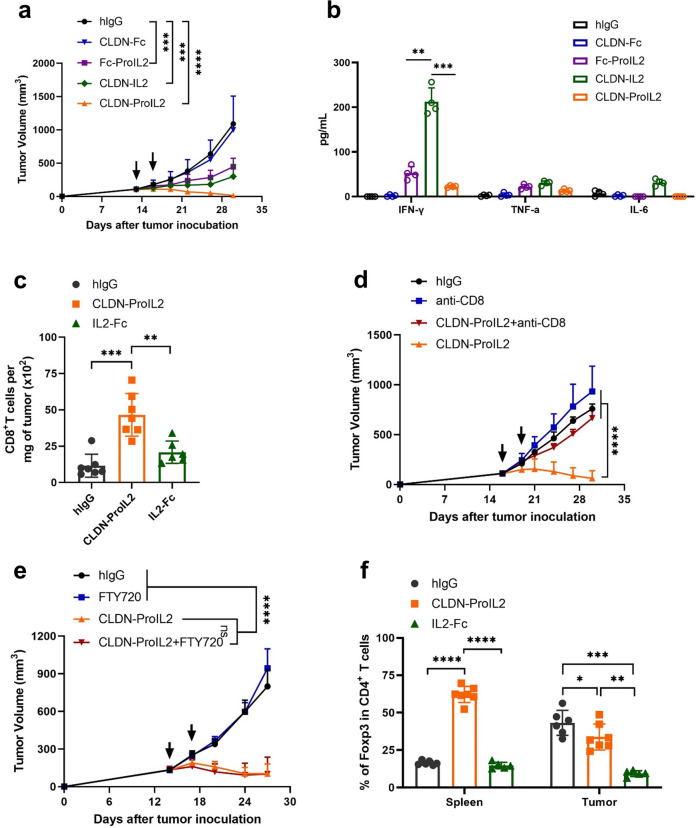
CLDN-ProIL2 targets CTLs inside TME while increases Treg cells in the peripheral with reduced toxicity and enhanced antitumor efficacy. **a**, **b** C57BL/6 J mice (*n* = 10/group) were subcutaneously inoculated with 5 × 10^5^ MC38-CLDN and treated with hIgG, CLDN-ProIL2 (60 μg) and equimolar doses of CLDN-Fc, Fc-ProIL2 or CLDN-IL2 by intraperitoneal injection on days 13 and 16 post-tumor inoculation. Tumor volume was measured twice a week (**a**). Serum was collected and isolated at 24 h post the first dose treatment. Cytometric Bead Array was used to quantify the amount of serum IFN-γ, TNF-α and IL-6 (**b**). **c**, **f** C57BL/6 J mice (*n* = 10/group) were subcutaneously inoculated with 5 × 10^5^ MC38-CLDN and treated with hIgG, CLDN-ProIL2 (60 μg) and equimolar doses of IL2-Fc by intraperitoneal injection on days 13 and 16 post-tumor inoculation. Four days after the second treatment, splenocytes and TILs were analyzed for the absolute number of CD8^+^ T cells per milligram of tumor tissues (**c**) and the frequency of Foxp3^+^CD4^+^ T cells (**f**). **d** MC38-CLDN tumor-bearing C57BL/6 J mice (*n* = 10/group) were treated with hIgG or CLDN-ProIL2 (60 μg) on days 15 and 18. Mice were intraperitoneally treated with anti-CD8 (200 μg/mouse) twice a week starting on day 14. Tumor volume was measured twice a week. **e** MC38-CLDN tumor-bearing C57BL/6 J mice (*n* = 10/group) were treated with hIgG or CLDN-ProIL2 (60 μg) on days 14 and 17. To block T cells migrating from lymph node into the tumor site, mice were administered with FTY720 every 2 days starting on day 13, and through the end of the experiment. Tumor volume was measured twice a week. Data are shown as means ± SEM. **a**–**f** is a pool of two independent experiments. The *P* value was determined by two-way ANOVA with Geisser-Greenhouse correction (**a**, **d** and **e**), one-way ANOVA with Tukey’s multiple comparisons test (**b**, **c** and **f**). * *P* < 0.05, ** *P* < 0.01, *** *P* < 0.001 and **** *P* < 0.0001, ns not significant

We next measured the tumor-targeting capacity of CLDN-ProIL2 using a bilateral tumor model and confirmed that CLDN-ProIL2 can substantially accumulate in CLDN positive tumor (Supplementary Fig. 5a, b) to deliver Pro-IL2. Encouragingly, CLDN-ProIL2 can induce much better tumor controls than Pro-IL2 in poorly immunogenic B16-CLDN tumors (Supplementary Fig. 5c) and MDA-MB231-CLDN tumor bearing humanized mice (Supplementary Fig. 5d). Nevertheless, the antitumor efficacy was not detected in CLDN negative tumors, demonstrating that tumor-targeting is required for the therapeutic effect of CLDN-ProIL2 (Supplementary Fig. 5e). Together, these results suggest that targeting tumors with Pro-IL2 by anti-CLDN is an important delivery strategy.

We discovered that the total number of CD8^+^ T cells in the tumor tissues were increased after CLDN-ProIL2 treatment (Fig. 1c). Then, we evaluated the contribution of innate and adaptive immunity for the therapeutic effect. Compared with WT mice, CLDN-ProIL2 failed to control tumor growth in *Rag1*^*−/−*^ mice but had remained anti-tumor effect in WT mice after NK cells depletion (Supplementary Fig. 6a, b), suggesting that T cells but not NK cells are required for the antitumor immune response. Indeed, the antitumor capability of CLDN-ProIL2 were completely abolished after CD8^+^ T cells depletion (Fig. 1d). To study if pre-existing TILs are sufficient to control tumor after treatment, FTY720, which can greatly block the trafficking of T cells was applied. The antitumor efficacy of CLDN-ProIL2 were not affected after FTY720 treatment (Fig. 1e), suggesting that pre-existing CD8^+^ T cells were sufficient and essential for tumor control.

Treg cells limit immune responses.^3^ CLDN-ProIL2 reduced the frequency of Treg cells in the tumor but unexpectedly increased it in the spleen (Fig. 1f). Such increase might contribute to minimized systemic toxicity. Interestingly, the frequency of CTLA4 and LAG3 in CD4^+^ T cells were decreased (Supplementary Fig. 7a, b). Moreover, the frequency of CD39^+^ cells in PD1^+^TIM3^+^CD8^+^ T cells were also decreased after CLDN-ProIL2 treatment (Supplementary Fig. 7c), suggesting that CLDN-ProIL2 can downregulate the expression of co-inhibitory molecules in the TME.

Tumor-specific T cells are required for the memory T cells formation and establishing systemic protective immunity against relapse.^4^ We observed tumor-specific CD8^+^ T cells (Supplementary Fig. 8a) were profoundly increased in the draining lymph nodes (dLNs) after CLDN-ProIL2 treatment. In addition, the percentage of PD1^+^TIM3^−^CD8^+^ T cells were increased whereas that of PD1^+^TIM3^+^ CD8^+^ T cells were decreased (Supplementary Fig. 8b), indicating that CLDN-ProIL2 reshaped the distribution of T cells in the TME to provoke antitumor immune response. Consistently, CLDN-ProIL2 significantly increased the percentage of IFN-γ-producing CD8^+^ T cells (Supplementary Fig. 8c, d). CLDN-ProIL2-cured mice can also eradicate re-challenged tumor cells (Supplementary Fig. 8e, f). These results suggest that protective immunological memory effectively generated in CLDN-ProIL2-treated mice.

Moreover, the frequency of proliferating CD8^+^ T cells (Supplementary Fig. 9a), the stem-like TCF1^+^TIM3^−^ CD8^+^ T cells (Supplementary Fig. 9b) and IFN-g producing CD8^+^ T cells (Supplementary Fig. 9c, d) within the tumor tissues were also increased after CLDN-ProIL2 treatment, suggesting CLDN-ProIL2 reshaped and reactivated intratumoral TILs. Meanwhile, FTY720 treatment didn’t block CLDN-ProIL2 mediated increase of PD1^+^TIM3^-^CD8^+^ T cells, the absolute number of PD1^+^TIM3^-^CD8^+^ T cells were still increased even after FTY720 plus CLDN-ProIL2 and CLDN-ProIL2 treatment (Supplementary Fig. 9e) compared to control group. Together, the data indicate that CLDN-ProIL2 can control tumors without newly entry T cells and suggest the treatment indeed can increase the function of intratumoral PD-1^+^TIM3^-^CD8^+^ TILs.

We next explored if CLDN-ProIL2 can generate systemic antitumor effects after local treatment in a bilateral tumor model (Supplementary Fig. 10a). Significant antitumor effects were observed in both treated and untreated tumors (Supplementary Fig. 10b). More importantly, all CLDN-ProIL2-cured mice can reject rechallenged MC38-CLDN tumor cells (Supplementary Fig. 10c), suggesting that local treatment of CLDN-ProIL2 in primary tumors can reactivate pre-existing TILs to form memory cells that control distal tumors.

Emerging evidence showed that neoadjuvant immunotherapy has emerged as a promising treatment against metastasis lesion.^5^ To explore if CLDN-ProIL2 can also serve as an effective neoadjuvant therapy to control metastasis. 4T1-CLDN tumor-bearing mice were preoperatively treated with CLDN-ProIL2 followed by surgical resection (Supplementary Fig. 10d). We observed that CLDN-ProIL2 and CLDN-ProIL2 plus surgery significantly reduced metastatic nodules (Supplementary Fig. 10e) and colony formation in the lung tissues (Supplementary Fig. 10f). Interestingly, the therapeutic efficacy was robustly abolished after FTY720 interference (Supplementary Fig. 10e, f), suggesting that dLNs play a critical role for CLDN-ProIL2 induced systemic antitumor immune response. Overall, our next generation CLDN-ProIL2 fusion protein which targeted CTLs inside TME while increased Treg cells in the peripheral for reduced toxicity, displayed an effective and safe profile with clinical translation potency.

## Supplementary information

supplemental data

## Data Availability

The data and materials used in the current study are available from the corresponding authors upon reasonable request.
