# Supplementary material for: Targeting tumor microenvironment with antibody-guided IL-2 pro-cytokine promotes and rejuvenates dysfunctional CD8+ T cells
Source: Signal Transduct Target Ther. 2023 Jul 12;8:268. doi: 10.1038/s41392-023-01463-y (PMC10336100; doi:10.1038/s41392-023-01463-y)
Supplement: Supplementary file 1 — supplemental data [file 41392_2023_1463_MOESM1_ESM.docx]

Supplementary Materials for

Targeting tumor microenvironment with antibody-guided IL-2 pro-cytokine promotes and rejuvenates dysfunctional CD8^+^ T cells

Xue Wang^1^, Longchao Liu^2,*^, Tao Yue^3^, Zhichen Sun^1,4^, Joonbeom Bae^1^, Kuo-Fu Tseng^3^, Anli Zhang^5^, Jian Qiao^1,*^, and Yang-Xin Fu^1,6,*^

Correspondence to: liulongchao@im.ac.cn (L.L), [Jian.Qiao@UTSouthwestern.edu](mailto:Jian.Qiao@UTSouthwestern.edu) (J.Q.) and [yangxinfu@tsinghua.edu.cn](mailto:yangxinfu@tsinghua.edu.cn) (Y.-X. F.)

**This file includes:**

Materials and Methods

Figures. S1 to S10

Table S1

**Materials and Methods**

**Mice**

Female (6-8 weeks old) C57BL/6J were purchased from Jackson Laboratory. *Rag1^−/−^* (B6.129S7-Rag1^tm1Mom^/J) mice on C57BL/6J background and NSG-SGM3 mice were purchased from UT Southwestern breeding core. All mice were maintained under speciﬁc pathogen-free conditions and animal experiments were conducted according to guidelines set by the Institutional Animal Care and Use Committee of the University of Texas Southwestern Medical Center.

**Cell lines and reagents**

MC38, B16, 4T1 and MDA-MB231 cell lines were purchased from American Type Culture Collection (ATCC) (Supplementary Table S1). MC38-CLDN, B16-CLDN, 4T1-CLDN and MDA-MB231-CLDN cell lines were obtained from lentiviral transduction of mouse derived CLDN18.2 gene. All these cells were cultured in 5% CO_2_ and maintained in Dulbecco’s modified Eagle’s medium (DMEM) supplemented with 10% heat-inactivated fetal bovine serum (FBS), 100 U/mL penicillin and 100 μg/mL streptomycin, and routinely tested for *Mycoplasma* contamination. Anti-IL-2Rβ (TM-Beta1), anti-CD8 (53-5.8) and anti-NK1.1 (PK136) were purchased from BioXCell. FcγRII/III blocking antibody (2.4G2) was produced in house. Goat anti-human IgG-HRP (sc-2453) was purchased from Santa Cruz. FTY720 was purchased from Selleckchem.

**Production of fusion proteins**

For CLDN-ProIL2 construction, the fragment of murine CLDN cDNA (heavy chain VH) was fused to the N-terminal of the whole variant Fc region (human IgG1), and IL2Rα carrying with MMPs cleavable substrate sequence coupled by wide-type IL-2 via a GGGGS linker was fused to the C-terminal of human IgG1 Fc region. The entire sequence and murine CLDN cDNA (light chain VL) were then cloned into the pEE6.4 vector (Lonza), respectively. For CLDN-IL2 construction, wide-type IL-2 was fused to the C-terminal of human IgG1 Fc region and the resting structures are the same as CLDN-ProIL2. For Fc-ProIL2 construction, IL2Rα carrying with MMPs cleavable substrate sequence coupled by wide-type IL-2 via a GGGGS linker was fused to the C-terminal of human IgG1 Fc region in the pEE6.4 vector (Lonza). Above fusion proteins were expressed via transient transfection of FreeStyle 293F cells and were purified using CaptivA^®^ Protein A Affinity Resin according to the manufacturer’s instructions (Repligen).

**In Vivo Imaging**

CLDN-ProIL2 was labeled with IRDye^®^800CW Dye according to the manufacturer’s instructions. MC38 (left flank) and MC38-CLDN (right flank) tumor-bearing NSG-SGM3 mice were intravenously injected with labeled CLDN-ProIL2 on day 8 after tumor inoculation. Fluorescence radiances was measured by LI-COR^®^ Pearl Trilogy imaging system at 0, 24, 48, 72, 96, 120 and 144 hours after injection.

**IL-2 reporter cell culture assay**

Various dilutions of fusion proteins were incubated with 5×10^4^ HEK-Blue^TM^ IL-2 reporter cells (InvivoGen) for 24 h according to the manufacturer’s instructions. Then 25 μL cell supernatant was added into a new 96-well plate and incubated with 75 μL Quanti-Blue reagent (InvivoGen) at 37°C for 2 h. The plate was read at 605-650 nm using the SPECTRO star Nano (BMG LABTECH).

**MMPs cleavage of CLDN-ProIL2 in vitro**

Recombinant human MMP2 (BioLegend), MMP9 (BioLegend) and MMP14 (R&D Sciences) were pre-activated respectively according to the manufacturer’s instructions. Cleavage assay buffer (pH 7.5) consisted of 50 mM Tris, 10 mM CaCl_2_, 150 mM NaCl and 0.05% (w/v) Brij 35. Cleavage condition based on our previous methods. Briefly, 100 ng hMMP2, 100 ng hMMP9 and 200 ng hMMP14 were incubated with 3 μg CLDN-ProIL2 in the cleavage assay buffer at 37 °C for overnight. Finally, fusion proteins solution were run on the SDS-PAGE gel.

**IFN-γ enzyme-linked immunosorbent spot assay (ELISPOT)**

MC38-CLDN tumor-bearing C57BL/6J mice were intraperitoneal treated with CLDN-ProIL2 (60 μg) on days 11 and 14. Four days post the last treatment, draining lymph nodes were collected and processed into single cell suspension, and re-suspended in RPMI 1640 medium supplemented with 10% heat-inactivated FBS, 100 U/mL penicillin and 100 μg/mL streptomycin. A total of 4×10^5^ cells/well were seeded into 96-well plate with irradiated MC38-CLDN tumor cells (4×10^4^) to stimulate the tumor-speciﬁc T cells for 48 h. IFN-γ production was measured using IFN-γ ELISPOT assay kit according to the manufacturer’s protocol (BD Biosciences). The visualized spots were enumerated with the CTL-ImmunoSpot^®^ S6 Analyzer (Cellular Technology Limited).

**Tumor growth and treatment**

A total of 4×10^5^ to 2×10^6^ of MC38, MC38-CLDN, B16-CLDN and 4T1-CLDN tumor cells were inoculated subcutaneously in the right flank of 6-8 week-old female mice. For the bilateral tumor model, 2.5×10^5^ MC38-CLDN (on the left) and 5×10^5^ MC38-CLDN (on the right) were inoculated in both flanks of C57BL/6J mice, respectively. Mice were randomly grouped according to tumor volume and were intraperitoneally (i.p.) treated with CLDN-ProIL2 or with equimolar doses of hIgG, CLDN-Fc, CLDN-IL2, Fc-ProIL2 and IL2-Fc on indicated time points. For IL-2 signaling blockade, 200 μg of anti-IL-2Rβ was i.p. injected on day 4 and was introtumoral (i.t.) injected on days 6 and 8. For depletion of different types of cells, a 200 μg dose of anti-CD8 (53-5.8) was i.p. injected 1 day before CLDN-ProIL2 treatment and every 3 days for two weeks after that. A 300 μg of anti-NK1.1 (PK136) was i.p. injected 1 day before CLDN-ProIL2 treatment and every 3 days for two weeks after that. Tumor volumes were measured twice a week and volume was calculated (length×width×height/2). To block lymphocytes trafficking, 25 μg FTY720 was i.p. injected 1 day before treatment initiation and10 μg was administrated every other day for 2 weeks to maintain the blockade.

**Humanized mouse tumor model**

Human cord blood samples were provided by UT Southwestern Parkland Hospital. Human CD34^+^ cells were purified by positive immunomagnetic selection with anti-human CD34 microbeads (Stemcell). 4-weeks-old NSG-SGM3 female mice were irradiated with 100 cGy (X-ray irradiation with X-RAD 320 irradiator) followed by i.v. injection of 1×10^5^ CD34^+^ cells. Twelve weeks after engraftment, the mice with over 50% human CD45^+^ cells reconstitution were used for experiments. Hu-CD34 mice were subcutaneously inoculated with 2×10^6^ MDA-MB231-CLDN cells. Mice were i.p. treated with hIgG or CLDN-ProIL2 on indicated dose and timepoints.

**Toxicity studies**

MC38-CLDN tumor-bearing mice were i.p. dosed with CLDN-ProIL2 (60 μg) or the equimolar doses of CLDN-IL2 on days 9 and 12 post tumor inoculation. Serum were isolated at 96 h after the first treatment for AST and ALT quantification. The body weight change of mice was monitored.

**Flow cytometry analysis**

Single cell suspensions of cells were incubated with anti-CD16/32 antibody (anti-FcγIII/II receptor, clone 2.4G2) for 15 min to block nonspecific binding and then stained with conjugated antibodies (1:200 dilution) for 30 min at 4 °C in the dark. Fixable viability dye eFluor^TM^ 506 was used to exclude dead cells. Foxp3, TCF1 and Ki67 was stained intracellularly using True-Nuclear^TM^ transcription factor buffer set (BioLegend) according to the manufacturer’s instructions. Antibodies against CD45 (30-F11), CD8 (53-6.7), CD4 (RM4-5), PD1 (29F.1A12), Foxp3 (MF-14), CD39 (Duha59), CTLA4 (UC10-4B9), LAG3 (C9B7W), Ki67 (16A8) and IL-2 (JES6-5H4) were purchased from Biolegend. Anti-CD3 (145-2C11) was purchased from BD Biosciences. Anti-TIM3 (RMT3-23) and IFN-g (XMG1.2) were purchased from eBioscience. TCF1 (C63D9) was purchased from CST. Tetramer for H-2Kb KSP (KSPWFTTL) was purchased from MBL. BD^TM^ Cytometric Bead Array (CBA) Mouse Th1/Th2/Th17 Cytokine Kit was used to measure the IFN-γ, IL-6 and TFN-α in the serum and tissues following the manufacturer’s protocol (BD Biosciences). The detailed information of antibodies was listed in supplementary Table. Data were collected on CytoFLEX flow cytometer (Beckman Coulter) and analyzed using FlowJo (Tree Star) software.

**Analysis of tumor clonogenic formation in 4T1-CLDN metastatic model**

4T1-CLDN tumor-bearing mice were i.p. treated with CLDN-ProIL2 (60 μg) on days 9, 12 and 15 post-tumor inoculation. Primary tumors were resected on day 19. Twenty-one days after surgery, lung tissues from 4T1-CLDN tumor-bearing or tumor-resected mice were excised and digested with 1 mg/mL Collagenase I (Sigma) and 0.5 mg/mL DNase I (Roche) at 37 °C for 45 min, and then passed through a 70 μm cell strainer to remove large pieces of undigested lung tissues. Lungs cells were resuspended in completed Dulbecco’s modified Eagle’s medium at a 1:500 dilution containing 10 μg/mL 6-thioguanine. After 7 days, cells were stained with crystal violet staining solution and colonies were counted.

**Statistical analysis**

All the experimental data analyses were performed with GraphPad Prism (GraphPad Software Inc., San Diego, CA) statistical software and shown as mean ± SEM. The P value was determined by two-way ANOVA with Geisser-Greenhouse's correction or one-way ANOVA with Tukey’s multiple comparisons test. Unpaired two-tailed t tests were used for other analysis. Significance was adjusted by the method described in the Figure legend and *P* < 0.05 was considered statistically signiﬁcant.

**Figure. S1**

**Figure. S1. CD8^+^ T cell and IL-2 expression level positively correlates with better survival in different human cancer types.**

**a**, **b** Cumulative survival in Adrenocortical Carcinoma (ACC), Breast Invasive Carcinoma (BRCA), Skin Cutaneous Melanoma (SKCM)-metastasis and Lung Adenocarcinoma (LUAD) patients according to CD8^+^ T cells infiltration (**a**) and IL-2 expression level (**b**) in TCGA database (top 25% vs. bottom 25%).

**Figure. S2 Insufficient IL-2 impairs the endogenous immune response and limits CD8^+^ TILs expansion.**

**a**-**e** MC38 bearing C57BL/6J mice (n = 10-12/group) were intraperitoneally injected with 200 μg of anti-IL2Rβ on day 4, and on days 6 and 8 intratumorally. Experimental scheme of MC38 tumor model (**a**). Four days after the last injection, tumor-infiltrating T cells were analyzed for the total number of T cells (**b**), the absolute number of CD8^+^ T cells (**c**) and the frequency of T cell subsets (**d**) by flow cytometry. Tumor volume was measured twice a week (**e**). Data are presented as mean ± SEM and a pool of two independent experiments. The *P* value was determined by two-way ANOVA with Geisser-Greenhouse correction (**e**) or two-tailed unpaired t test (**b-d**). ** *P* < 0.01, *** *P* < 0.001 and *****P* < 0.0001.

**Figure. S3 CLDN-ProIL2 activity is effectively shielded by IL2Rα *in vitro*.**

**a** Schematic diagram of the anti-CLDN-IL2Rα-MMPs-IL2 homodimer (CLDN-ProIL2). **b** Binding of CLDN-Fc and CLDN-ProIL2 at different doses to MC38-CLDN cell lines was assessed via flow cytometry. **c** Binding of CLDN-ProIL2 at different doses to HEK-Blue^TM^ IL-2 reporter cells was assessed via flow cytometry. **d** Functional activity of IL2-Fc, CLDN-ProIL2 and CLDN-ProIL2 that have been incubated with human MMP2, MMP9, and MMP14 for overnight was measured by using HEK-Blue^TM^ IL-2 reporter cell assay. Data are shown as mean ± SEM from a representative experiment.

**Figure. S4 CLDN-targeting delivery of ProIL2 reduces systemic toxicity *in vivo.***

**a, b** C57BL/6J mice (n = 10/group) were subcutaneously inoculated with 5×10^5^ MC38-CLDN and treated with hIgG, CLDN-ProIL2 (60 μg) and equimolar doses of CLDN-IL2 by intraperitoneal injection on days 13 and 16 post-tumor inoculation. Bodyweight (**a**) and survival curve (**b**) were monitored at indicated time points. **c**, **d** C57BL/6J mice (n = 3-5/group) were subcutaneously inoculated with 5×10^5^ MC38-CLDN tumor cells and treated with hIgG, CLDN-ProIL2 (60 μg) and equimolar doses of CLDN-IL2 on day 12. Peripheral blood was collected at 72 h after the first treatment to measure the percentage of NK cells (**c**) and CD8^+^ T cells (**d**). **e**, **f** C57BL/6J mice (n = 5-7/group) were subcutaneously inoculated with 5×10^5^ MC38-CLDN tumor cells and treated with hIgG, CLDN-ProIL2 (60 μg) and equimolar doses of CLDN-IL2 on days 9 and 12 post-tumor inoculation. Serum were collected at 96 h after the first treatment. The serum of ALT (**e**) and AST (**f**) were quantified.

**Figure. S5 CLDN-ProIL2 enhanced the antitumor immune response of ProIL2 through tumor**-**targeting delivery.**

**a**, **b** NSG-SGM3 mice (n = 8/group) were inoculated with 1×10^6^ MC38-CLDN tumor cells in the right flank and 0.6×10^6^ MC38 tumor cells in the left flank on day 0 and then intravenously treated with 2 μg IRDye 800CW-labeled CLDN-ProIL2 on day 8, and mice without treatment served as control. Assessment of the tissue distribution of CLDN-ProIL2 by whole-body fluorescence imaging (**a**) and isolated tumor tissues after 24 h treatment (**b**). **c** C57BL/6J mice (n = 10/group) were subcutaneously inoculated with 7×10^5^ B16-CLDN and treated with hIgG, CLDN-ProIL2 (60 μg) and equimolar doses of Fc-ProIL2 by intraperitoneal injection on days 8 and 10 post-tumor inoculation. Tumor volume was measured twice a week. **d** NSG-SGM3 mice (n = 8/group) were adoptively transferred with 1×10^5^ human CD34^+^ HSCs. Twelve weeks later, 2×10^6^ MDA-MB231-CLDN cells were inoculated followed by intraperitoneal treatment of hIgG or CLDN-ProIL2 for the first dose on day 33 (30 μg), and continuous treatment on days 36 (60 μg), 38 (60 μg), 41 (60 μg), 43 (60 μg) and 45 (60 μg). Tumor volume was measured twice a week. **e** C57BL/6J mice (n = 8-10/group) were subcutaneously injected with 5×10^5^ MC38 and treated with equimolar doses of hIgG and CLDN-ProIL2 (30 μg) by intraperitoneal injection on days 11 and 14 post-tumor inoculation. Tumor volume was measured twice a week. Data are shown as means ± SEM. **c** and **e** is a pool of two independent experiments. The *P* value was determined by two-way ANOVA with Geisser-Greenhouse correction (**c-e**). **** *P* < 0.0001, ns, not significant.

**Figure. S6 CLDN-ProIL2 mediated antitumor efficacy depends on the pre-existing CD8^+^ TILs.**

**a** *Rag1*^-/-^ and C57BL/6J mice (n = 8-10/group) were subcutaneously inoculated with 5×10^5^ MC38-CLDN and treated with hIgG or CLDN-ProIL2 (30 μg) intraperitoneally on days 8 and 11 post-tumor inoculation. Tumor volume was measured twice a week. **b** MC38-CLDN tumor-bearing C57BL/6J mice (n = 10/group) were intraperitoneal treated with hIgG or CLDN-ProIL2 on days 10 (60 μg) and 13 (30 μg). Mice were intraperitoneally treated with anti-NK1.1 (300 μg/mouse) on day 9 and twice a week for two weeks. One of two representative experiments is shown in (**b**). The *P* value was determined by two-way ANOVA with Geisser-Greenhouse correction (**a** and **b**), **** *P* < 0.0001, ns, not significant.

**Figure. S7 CLDN-ProIL2 decreased the expression of immune co-inhibitory molecules in MC38-CLDN tumor-bearing mice.**

**a-c** MC38-CLDN tumor-bearing C57BL/6J mice (n = 4/group) were intraperitoneally treated with hIgG or CLDN-ProIL2 (60 μg) on day 12 post-tumor inoculation. 48 h after the first treatment, TILs were analyzed for the frequency of CTLA4 in CD4^+^ T cells (**a**), frequency of LAG3 in CD4^+^ T cells (**b**) and the frequency of CD39^+^PD1^+^TIM3^+^ , CD39^+^ PD1^+^TIM3^-^ and CD39^+^ PD1^-^TIM3^-^ in CD8^+^ T cell subsets (**c**) in tumor tissues by flow cytometry. The P value was determined by one-way ANOVA with Tukey’s multiple comparisons test. ** P < 0.01, ns, not significant.

**Figure. S8 CLDN-ProIL2 reshapes a distinct immuno-phenotypic profile in tumor-bearing mice**.

**a-d** MC38-CLDN tumor-bearing C57BL/6J mice (n = 5-7/group) were intraperitoneally treated with hIgG, CLDN-ProIL2 (60 μg) and equimolar doses of IL2-Fc on days 9 and 11 post-tumor inoculation. Four days after the second treatment, dLNs and TILs were analyzed for the frequency of T cells tetramer^+^ T cells in CD3^+^ T cells via a H-2Kb KSP (KSPWFTTL) tetramer (**a**) and the frequency of PD1^-^TIM3^-^, PD1^+^TIM3^-^ and PD1^+^TIM3^+^ CD8^+^ T cell subsets in tumor tissues (**b**) by flow cytometry. Cells from dLNs were collected and re-stimulated with irradiated MC38-CLDN for 48 h. IFN-γ producing cells were detected by ELISPOT assay. The representative spots (**c**) and quantification data are shown in (**d**). **e** MC38-CLDN tumor-bearing C57BL/6J mice were intraperitoneally treated with hIgG or CLDN-ProIL2 (60 μg) on days 13 and 16, respectively. Sixty days after tumor inoculation, previously cured mice were re-challenged with 8-fold higher (4×10^6^) MC38-CLDN tumor cells on the opposite flank and tumor growth was compared to naïve control mice. Data are presented as mean ± SEM from a representative experiment. **f** B16-CLDN tumor-bearing C57BL/6J mice were intraperitoneally treated with hIgG, CLDN-ProIL2 (60 μg) and equimolar doses of Fc-ProIL2 by intraperitoneal injection on days 8 and 10, respectively. Sixty days after tumor inoculation, previously cured mice were re-challenged with 3-fold higher B16-CLDN tumor cells on the opposite flank and tumor growth was compared to naïve control mice. The *P* value was determined by two-way ANOVA with Tukey’s multiple comparisons test (**e** and **f**) or one-way ANOVA with Tukey’s multiple comparisons test (**a**, **b** and **d**). * *P* < 0.05, ** *P* < 0.01, *** *P* < 0.001 and **** *P* < 0.0001, ns, not significant.

**Figure. S9 CLDN-ProIL2 amplified and enhanced the function of PD1^+^TIM3^-^CD8^+^ T cells.**

**a, b** MC38-CLDN tumor-bearing C57BL/6J mice (n = 4-6/group) were intraperitoneally treated with hIgG or CLDN-ProIL2 (60 μg) on day 12. 24 h after the first treatment, TILs were analyzed for the frequency of Ki67^+^CD8^+^ T cells (**a**) and the frequency of TCF1^+^ TIM3^-^ in CD8^+^ T cells (**b**). **c, d** MC38-CLDN tumor-bearing C57BL/6J mice (n = 4/group) were intraperitoneally treated with hIgG or CLDN-ProIL2 (60 μg) on day 11 post-tumor inoculation. Mice were treated with Brefeldin A (BFA) by intravenously injection 250 μg, and then 6 h later tumor tissues were harvested. Experimental scheme of BFA experiment (**c**) and TILs were analyzed for the frequency of IFN-g in CD8^+^ T cells (**d**) by flow cytometry. **e** MC38-CLDN tumor-bearing C57BL/6J mice (n = 5-7/group) were treated with hIgG or CLDN-ProIL2 (60 μg) on days 14 and 17. To block T cells migrating from lymph node into the tumor site, mice were intraperitoneally treated with FTY720 (25 μg) starting on day 13 and were treated with 10 μg every other day through the end of the experiment. Four days after the second treatment, TILs were analyzed for the absolute number of PD1^+^TIM3^-^CD8^+^ T cells per milligram of tumor tissues by flow cytometry. P value was determined by two-tailed unpaired t test. * P < 0.05, ** P < 0.01, *** P < 0.001.

**Figure. S10 CLDN-ProIL2 treatment can elicit systemic antitumor immune responses and control metastasis formation.**

**a**, **b** C57BL/6J mice were subcutaneously inoculated with MC38-CLDN tumor cells (n = 8/group) on the right flank (5×10^5^, treated tumor) on day 0 and the left flank (2.5×10^5^, untreated tumor) on day 2. Experimental scheme of the bilateral tumor model (**a**). The right tumor was intratumorally treated with hIgG or CLDN-ProIL2 (15 μg) on days 11 and 13. Both treated and untreated tumor growth were monitored twice a week (**b**). **c** Tumor-bearing mice cured by CLDN-ProIL2 or naïve control mice were subcutaneously inoculated with 5×10^6^ MC38-CLDN tumor cells at a secondary site, and tumor growth were monitored twice a week. **d**-**f** Mice with 4T1-CLDN tumor cells (n = 10-15/group) were intraperitoneally treated with hIgG or CLDN-ProIL2 (60 μg) on days 9, 12 and 15 post-tumor inoculation. Mice were intraperitoneally treated with FTY720 (25 μg) on day 8 and were treated with 10 μg every other day for 18 days maintenance. Experimental design (**d**), on day 19 post-tumor inoculation, tumors were surgically removed from tumor-bearing mice. Forty days post-tumor inoculation, the remaining mice were euthanized and metastatic nodules in the thoracic cavity were counted (**e**), meanwhile, lungs were extracted, digested in collagenase/DNAase, and resuspended as single cells. Resuspended cells were plated at a 1:500 ratio with 6-thioguanine selection medium, then the number of colonies were counted 7 days post seeding (n = 3 experiments, total 10 individual mice for hIgG treatment group, 8 for Surgery, 8 for CLDN-ProIL2 and CLDN-ProIL2/Surgery, 9 for FTY720/CLDN-ProIL2/Surgery) (**f**). Data are shown as means ± SEM. **b** is a pool of two independent experiments. The *P* value was determined by two-way ANOVA with Geisser-Greenhouse correction (**b** and **c**) or one-way ANOVA with Tukey’s multiple comparisons test (**e** and **f**). **** *P* < 0.0001.

**Table. S1**

| **REAGENT or RESOURCE** | **SOURCE** | **IDENTIFIER** |
| --- | --- | --- |
| Antibodies | | |
| Anti-mouse CD45 (Flow cytometry, 30-F11) | BioLegend | Cat# 103126 |
| Anti-mouse CD3 (Flow cytometry, 145-2C11) | BD Biosciences | Cat# 564379 |
| Anti-mouse CD8 (Flow cytometry, 53-6.7) | BioLegend | Cat# 100730 |
| Anti-mouse CD4 (Flow cytometry, RM4-5) | BD Biosciences | Cat# 550954 |
| Anti-mouse PD-1 (Flow cytometry, 29F.1A12) | BioLegend | Cat# 135224 |
| Anti-mouse TIM-3 (Flow cytometry, RMT3-23) | eBioscience | Cat# 25587008 |
| Anti-mouse Foxp3 (Flow cytometry, MF-14) | BioLegend | Cat# 126408 |
| Anti-mouse CTLA4 (Flow cytometry, UC10-4B9) | BioLegend | Cat# 106314 |
| Anti-mouse LAG3 (Flow cytometry, C9B7W) | BioLegend | Cat# 125210 |
| Anti-mouse TCF1 (Flow cytometry, C63D9) | CST | Cat# 6444S |
| Anti-mouse Ki67 (Flow cytometry, 16A8) | BioLegend | Cat# 652404 |
| Anti-mouse CD39 (Flow cytometry, Duha59) | BioLegend | Cat# 143809 |
| Anti-mouse IFN-g (Flow cytometry, XMG1.2) | eBioscience | Cat# 17-7311-82 |
| Anti-mouse IL-2 (Flow cytometry, JES6-5H4) | BioLegend | Cat# 503806 |
| Anti-CD16/32 (clone 2.4G2) | In house | N/A |
| Granzyme B (QA16A02) | BioLegend | Cat# 372208 |
| Fixable Viability Dye eFluor™ 506 | Thermo Fisher | Cat# 65-0866-18 |
| H-2Kb MuLV p15E Tetramer-KSPWFTTL | MBL | Cat# TB-M507-1 |
| Goat anti-Human IgG (H+L)-HRP | Santa Cruz | Cat# sc-2453 |
| AffiniPure Goat Anti-Human IgG, Fcγ fragment  specific | Jackson ImmunoReseach | Cat# 109-055-098 |
| InVivoMAb anti-mouse CD8 (53-5.8) | BioXCell | Cat# BE |
| InVivoMAb anti-mouse NK1.1 (PK136) | BioXCell | Cat# BE0036 |
| Bacterial and Virus Strains | | |
| N/A |  |  |
| Biological Samples | | |
| N/A |  |  |
| Chemicals, Peptides, and Recombinant Proteins | | |
| FTY720 (hydrochloride) | Selleckchem | Cat# S5002 |
| 6-Thioguanine, 98%, Alfa Aesar | Thermo Fisher | Cat# AAB2128003 |
| Dulbecco’s Modified Eagle’s Medium | Sigma- Aldrich | Cat# D6429 |
| RPMI-1640 | Sigma- Aldrich | Cat# R8758-500ML |
| Collagenase type I | Sigma | Cat# C0130 |
| QUANTI-Blue^TM^ | InvivoGen | Cat# rep-qbs |
| DNase I | Roche | Cat# 11284932001 |
| Recombinant Human MMP-2 (carrier-free) | BioLegend | Cat# 554302 |
| Recombinant Human MMP-9 (carrier-free) | BioLegend | Cat# 550502 |
| Recombinant Human MMP-14/MT1-MMP  (NS0-expressed) Protein, CF | R&D Systems | Cat# 918-MPN-  010 |
| Critical Commercial Assays | | |
| BD Mouse IFN-γ ELISPOT Sets | BD Biosciences | Cat# 551083 |
| True-Nuclear™ Transcription Factor Buffer Set | BioLegend | Cat# 424401 |
| BD™ Cytometric Bead Array (CBA) Mouse Th1/Th2/Th17 Cytokine Kit | BD Biosciences | Cat# 560485 |
| Deposited Data | | |
| N/A |  |  |
| Experimental Models: Cell Lines | | |
| MC38 | ATCC | N/A |
| B16 | ATCC | Cat# CRL-6475 |
| 4T1 | ATCC | Cat# CRL-2539 |
| MDA-MB231 | ATCC | Cat# HTB-26™ |
| 293T | ATCC | Cat# CRL-3216 |
| FreeStyle^TM^ 293-F | Thermo Fisher | Cat# R79007 |
| HEK-Blue^TM^ IL-2 reporter cells | InvivoGen | Cat# hkb-il2 |
| Experimental Models: Organisms/Strains | | |
| C57BL/6J | Jackson Laboratory | Strain# 000664 |
| NSG-SGM3 | Jackson Laboratory | Strain# 017581 |
| Oligonucleotides | | |
| N/A |  |  |
| Recombinant DNA | | |
| Plasmid: pMD2.G | In house | N/A |
| Plasmid: psPAX2 | In house | N/A |
| Plasmid: pCDH-mouse CLDN18.2 | In house | N/A |
| Plasmid: pEE6.4-VH CLDN18.2-Fc-IL2Ra-MMP-IL2 | This paper | N/A |
| Plasmid: pEE6.4- VH CLDN18.2-Fc-IL2 | This paper | N/A |
| Plasmid: pEE6.4-IL2-Fc | This paper | N/A |
| Plasmid: pEE6.4-VL CLDN18.2 | This paper | N/A |
| Plasmid: pEE6.4-Fc-IL2Ra-MMP-IL2 | This paper | N/A |
| Software and Algorithms | | |
| GraphPad Prism software 7.0 | GraphPad Software, Inc. | <https://graphpad.com/scientific-software/prism/> |
| CTL-ImmunoSpot® S6 Analyzer | Cellular Technology Limited | <http://www.immunospot.com/ImmunoSpot-analyzers> |
| CytExpert | Beckman Coulter, Inc | <https://www.beckman.com/coulter-flow-cytometers/cytoflex/cytexpert> |
| BD FACSChorus™ Software | BD Biosciences | https://www.bdbiosciences.com/en-us/instruments/research-instruments/research-software/flow-cytometry-acquisition/facschorus-software |
| FlowJo | Tree Star Inc. | <https://www.flowjo.com/solutions/flowjo> |
| Timer | Li et al., 2017 | https://cistrome.shinyapps.io/timer/ |
| Other | | |
| N/A |  |  |
